# Defining localities of inadequate treatment for childhood asthma: A GIS approach

**DOI:** 10.1186/1476-072X-5-3

**Published:** 2006-01-17

**Authors:** Ronit Peled, Haim Reuveni, Joseph S Pliskin, Itzhak Benenson, Erez Hatna, Asher Tal

**Affiliations:** 1 Department of Health Systems Management, Ben-Gurion University of the Negev, Beer-Sheva, Israel; 2 Pediatrics Department, Soroka University Medical Center, Beer-Sheva Israel; 3 Department of Industrial Engineering and Management, Ben-Gurion University of the Negev, Beer-Sheva, Israel; 4 Department of Geography, Tel Aviv University, Tel Aviv, Israel

## Abstract

**Background:**

The use of Geographic Information Systems (GIS) has great potential for the management of chronic disease and the analysis of clinical and administrative health care data. Asthma is a chronic disease associated with substantial morbidity, mortality, and health care use. Epidemiologic data from all over the world show an increasing prevalence of asthma morbidity and mortality despite the availability of effective treatment. These facts led to the emergence of strategies developed to improve the quality of asthma care.

**The objective:**

To develop an efficient tool for quality assurance and chronic disease management using a Geographic Information System (GIS).

**Geographic location:**

The southern region of Israel. January 1998 – October 2000.

**Databases:**

Administrative claims data of the largest HMO in Israel: drug dispensing registry, demographic data, Emergency Room visits, and hospitalization data bases.

**Methods:**

We created a list of six markers for inadequate pharmaceutical treatment of childhood asthma from the Israeli clinical guidelines. We used this list to search the drug dispensing registry to identify asthmatic children who received inadequate treatment and to assess their health care utilization and bad outcomes: emergency room visits and hospitalizations. Using GIS we created thematic maps on which we located the clinics with a high percentage of children for whom the treatment provided was not in adherence with the clinical guidelines.

**Results:**

81% of the children were found to have at least one marker for inadequate treatment; 17.5% were found to have more than one marker. Children with markers were found to have statistically significant higher rates of Emergency Room visits, hospitalizations and longer length of stay in hospital compared with children without markers. The maps show in a robust way which clinics provided treatment not in accord with the clinical guidelines. Those clinics have high rates of Emergency Room visits, hospitalizations and length of stay.

**Conclusion:**

Integration of clinical guidelines, administrative data and GIS can create an efficient interface between administrative and clinical information. This tool can be used for allocating sites for quality assurance interventions.

## Introduction

### GIS

The use of information technologies such as the Geographic Information Systems (GIS) has great potential for the management of chronic disease and the analysis of clinical and administrative health care data.

Geographic mapping of health phenomena has been well-known since the dot maps created by Snow in 1855 which allocated cholera outbreaks in London, England. In the last decades, the use of geographic mapping has been expanded especially since the development of GIS technologies which comprise data analysis through spatial strategies and a visual presentation of the information [1-3]. The impact of GIS has been widely felt in all fields that use geographic information – resource management [4], land-use planning [5], pollution [6-8], transportation and utilities, marketing, geosciences and planning of health care facilities[9,10].

Burrough [11] identified five basic functional components in GIS: (1) data acquisition and data verification; (2) data storage and database management; (3) data transformation and analysis; (4) data output and presentation; and (5) user interface.

One of the more powerful features of GIS is the ability to link several data bases such as demographic, clinical, and billing systems, and create a high resolution demonstration of the spatial distribution and or behavior of the phenomenon. Therefore, the use of GIS in health related studies is emerging as an important tool in health care planning, quality assurance, and research.

GIS was used by researchers to analyze spatial patterns of cancer mortality in China [12], identifying high levels of lead exposure in children [13], defining localities for the management of primary health care in England [14], mapping and analyzing rates and distribution of child abuse in order to allocate special services [15]as well as many other applications.

### Asthma

The epidemiology of asthma has drawn much attention in the past two decades since data from the United States, as well as from around the world, showed an increasing prevalence of asthma morbidity and mortality despite the availability of effective treatment [16]. Asthma is a chronic disease associated with substantial morbidity, mortality, and health care use. Between 1980 and 1994, the self-reported prevalence of asthma increased 75% among all race, sex, and age groups in every region of the United States. Although an estimated 14.6 million persons had asthma in the United States in 1996, more recent studies have suggested a plateau of the prevalence of the disease [17]. Asthma is recognized nowadays as the most common chronic disease for children and adults and has become an economic burden to patients, their families, health care providers, and society.

Epidemiologic literature on asthma describes large geographic variations in asthma outcomes [18,19]. These variations were found among states [19], among counties within states [20], among cities [18]and even among neighborhoods within cities [21]. These studies raised important questions as to the role that quality of health care plays in contributing to these geographic variations. Understanding the link between quality of care and poor clinical outcomes for asthma patients led to the emergence of strategies developed to improve the quality of asthma care.

### Clinical guidelines and quality of care

Although there are many ways to approach quality of care improvement, most of them fall into two general areas: professional knowledge relating to diagnosis and treatment, and knowledge for improvement related to the design of the delivery of care [22]. The professional knowledge is presented in clinical guidelines for asthma treatment, which are a summary of clinical evidence and expert opinion. These clinical guidelines can play a major role in the quality improvement process and in the design of the delivery of care. Thus, an adequate implementation of the clinical guidelines became crucial for health care organizations who maintain a quality assurance process, since it was well documented that patients who received treatment in adherence to the clinical guidelines achieved the treatment goals [23,24].

### The objective

The objective of this study was to develop a tool, using GIS technology, to locate inappropriate treatment and poor treatment outcomes for children with asthma, as a result of inadequate implementation of the clinical guidelines. We hypothesized that in a world of high quality computerized data, such a tool could be useful for decision analysis while planning health care delivery and maintaining quality assurance procedures.

## Methods

### Geographic location

The study was conducted in the southern region of Israel using data sets of Clalit Health Services (CHS), the largest HMO in Israel, through which 770,000 members, including 143,000 infants, are insured.

### Study period

January 1998 – October 2000.

### Information sources

We used the three parts of the CHS claims data sets: 1. Drug dispensing registry which included patient ID, name and serial number of the drug dispensed and date of dispensing; 2. Demographic data which included: ID, date of birth, address, name and address of affiliated clinic; 3. Health care utilization data sets which included: Emergency Room (ER) visits to pediatric and internal medicine ER wards; and hospitalizations in pediatric, internal medicine, ear nose throat (ENT), and Intensive Care Unit (ICU) wards. Hospitalizations and ER visits were considered bad treatment outcomes. Diagnoses in admission and/or while visiting the ER could not be obtained, as we could not be sure that these admissions and/or ER visits were related to Asthma. However we assume that children with asthma who receive an adequate pharmaceutical treatment are less susceptible.

### Definition of inappropriate treatment

The Israeli clinical guidelines for drug therapy for childhood asthma[25] were translated into a list of six indicators for inadequate treatment defined as "markers" (Table 1). These indicators were agreed by an expert consensus including pediatricians and respiratory specialists.

**Table 1:** Markers for inadequate treatment of asthma

**Marker Number**	**Description**
**1**	More than 55 days of relievers' treatment without treatment with controllers.
**2**	Dispensation of more than one long-acting agonist with no other treatment.
**3**	High frequency of drug treatment changes.
**4**	More than 15 dispensations of controllers per year (excluding antileukotriene).
**5**	High ratio of reliever to controller prescriptions.
**6**	More than 30 tablets of Beta-agonist per year.

### Study population

The study population included 6,776 children 3 to 17 years of age insured by CHS in the southern district of Israel for whom at least two dispensations for asthma drugs were registered during 1998. Children with other chronic diseases such as Cystic Fibrosis, or children with unreasonable drug utilization (10 times the district average) were excluded.

### Validation

In order to ensure that the children who were identified as eligible for the study do have asthma, we created a randomized sample of 300 families from the study population, who were interviewed by phone and were asked if the child was diagnosed by a physician as having asthma. Two hundred and seventy-one (90.3%) families agreed to be interviewed. Two hundred and forty eight families (91.5%) reported that the child was diagnosed by a physician as having Asthma.

## Constructing the files

### Drugs

In order to unify the drug dispensations, we translated each dispensation into treatment days according to the recommended dosage. For example: Budicort 50 mcg/1 Inhal = 200 units, (recommendation: 3 times a day) = 65 treatment days.

### Health care utilization

Using these files, we computed the number of utilizations per child during the study period. All data sets were combined into one file where each record, for each child participating in the study, included the following variables: age, sex, affiliated clinic, treatment days for each drug, number of ER visits, number of hospitalizations and length of stay, number of GP and specialist visits and number of imaging tests.

### Searching for inappropriate treatment

Using the markers list (Table 1) we electronically searched the file and marked the subjects who were found to have one or more markers, and the marker type. For example, if a record contained 65 days of relievers use without any use of controller, this record was signed as having "marker no.1". By the end of the process the system calculated the frequency of each marker.

### GIS mapping

In the first step, we geocoded all study subjects according to their clinic affiliation. We managed to geocode 100% of our study population. In the second step, we created thematic maps for each study area. Each map describes the distribution of patients with or without inappropriate treatment by clinic. Clinics with 10% above the regional average for inappropriate treatment were "flagged." In the third step, we mapped the adverse outcomes: emergency room (ER) visits and hospital admissions, also by clinic affiliation. These maps describe the distribution of patients who have or have not visited an ER and were or were not admitted to a hospital. These maps demonstrate the association between inappropriate treatment and adverse outcomes. MapInfo 6.5 software was used for the GIS mapping.

### Statistical Methods

In order to compare ER visits, number of hospitalizations and length of stay between children with or without markers for inadequate treatment, and because the means were not normally distributed, we used a non-parametric, the Mann Whitney U, test.

## Results

6,776 children from 146 CHS clinics were identified as eligible for participating in the study, their mean age was 7.6 years, 57.8% were boys (Table 2). Eighty-one percent of the children were found to have at least one marker for inadequate treatment; 17.5% were found to have more than one marker. The most common markers were marker number 5 (high ratio of reliever to controller prescriptions) and marker number 1 (more than 55 days of relievers treatment without treatment with controllers). Patients with markers had significantly more ER visits and hospitalizations, and were hospitalized for a longer period compared with patients without markers (Table 3).

**Table 2:** Study population characteristics

	**n**	**%**
**Boys**	3,919	57.8
**Girls**	2,857	42.2
**Total**	6,776	100.0
**Median Age**		6.0
**Mean Age**		7.6
**S.D**		4.0
**Range**		3–17

**Table 3:** Outcomes: mean (standard deviations) for ER visits, hospital admissions and length of stay by marker for the study period (34 months).

**Outcomes**	**With Markers n = 5,498**	**Without Markers 1,278 n =**	**p**
***No. of ER visits***			
**Mean**	1.5 (3.4)	1.0 (2.2)	
**Median**	1.0	0.00	
**Min.-Max.**	0–30	0–15	
**Mean Rank**	3423.46	3238.10	0.000

***No***. ***Hospitalizations***			
**Mean**	0.4 (1.5)	0.2 (0.1)	
**Median**	0.00	0.00	
**Min.-Max.**	0–27	0–14	
**Mean Rank**	3406.86	3309.50	0.004

***Length of stay (Days)***			
**Mean**	1.4 (7.3)	0.7 (3.3)	
**Median**	0.00	0.00	
**Min.-Max.**	0–150	0–54	
**Mean Rank**	3406.70	3310.21	0.005

**Figure 1:**
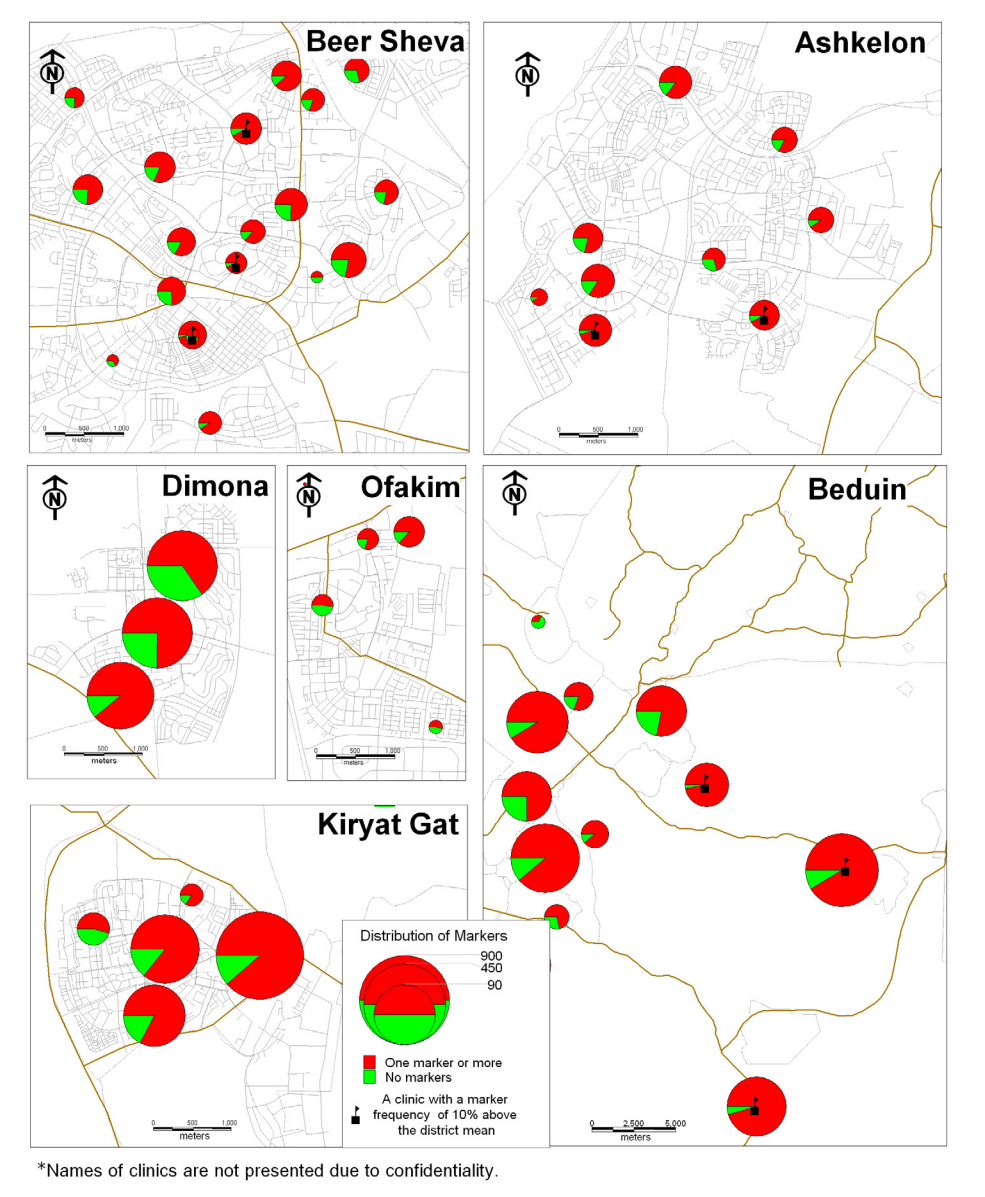
Map 1: Ditribution of markers.

**Figure 2:**
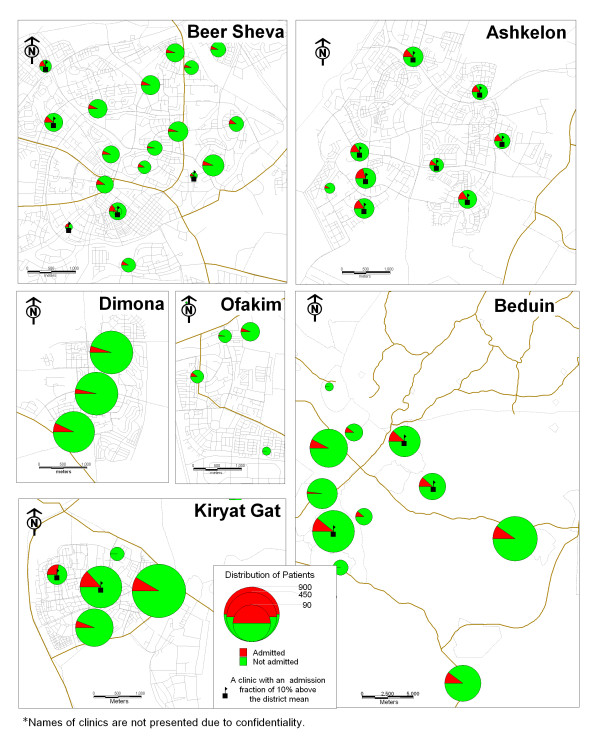
Map 2: Hospital admissions.

**Figure 3:**
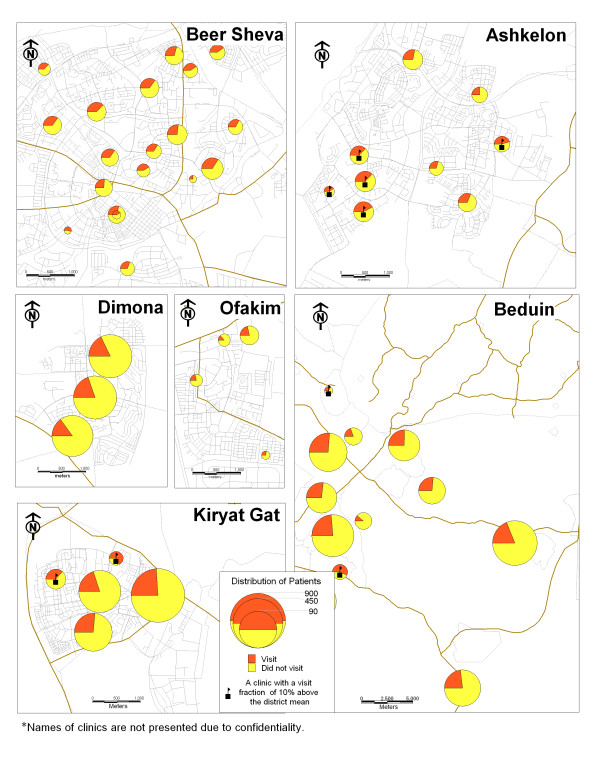
Map 3: Emergency room visits.

The results of the GIS mapping show that markers for inadequate treatment are more prevalent in the clinics which provide health care services for Bedouin citizens. In this area, in one-third of the clinics the rate of children with markers is ten percent higher than the regional average. In the city of Ashkelon, one-quarter of the clinics had higher rates of markers than the regional average (Figure 1). Figures 2 and 3 demonstrate the association between inadequate treatment and adverse outcomes. The same clinics with a high percentage of markers also have high percentages of ER visits and hospital admissions.

## Discussion

Chronic disease is the major component of the utilization of health services in developed countries. The increase in the number of chronic patients in industrialized countries continues to rise steadily and with it the need of the health care systems to allocate resources accordingly. One of the ways in which the priority of resource allocation can be changed is by improving the standard of care. From this premise, guidelines and other quality assurance techniques were developed which strive to achieve uniformity of treatment and constant improvement in its quality, while decreasing costs.

This study presents a unique model for the management of a chronic illness using a Geographic Information System (GIS). This model was tested on the management of childhood asthma in the southern region of Israel. The motivation behind this study was to respond to the following problems: (1) The rise in asthma morbidity rates among children and adults for which the treatment is dispensed by family practitioners and specialists; (2) The difficulties in implementing clinical guidelines; and (3) The geographic variations in asthma outcomes as documented world-wide [18-21] and in Israel [26,27]. The maps we created demonstrate the geographic variations in outcome parameters: ER visits and hospitalization rates, and can be attributed to the lack of adequate treatment. In other words, we can see that in those clinics where treatment is not provided in accordance with the guidelines, the rates for adverse outcomes are higher.

The information presented by the maps can be also presented in traditional ways such as tables produced from sophisticated statistical analyses, but there is no doubt that these maps are a better way to present the phenomena.

While using GIS technology, it is very important that the geographic component (e.g., address, zip code, clinic affiliation, etc.) in the data base is accurate, otherwise the geocode process will fail.

The limitation of this model lies in the fact that we used drug dispensing registries. We used these data to identify our study population and to identify inadequate treatment. In previous studies [28-30], it was suggested that it is appropriate to use the drug dispensing data in order to identify patients in general, as well as for asthma patients specifically, even if ranking the patients according to the severity of the disease is required. However, in our study we do not have information on: [1] whether the patient actually purchased the medication prescribed by the physician. There is a possibility that the physician issued a prescription but the patient decided not to follow the treatment prescribed. If this bias exists, the practice will have been misjudged in these cases. We think this bias can be considered as a very mild one; and, [2] we do not have information about patients' compliance with the treatment: the patient purchased the drug but did not actually take it. If this bias exists, we may over-estimate and misjudge the treatment outcomes, especially as a result of inadequate treatment.

A lack of access to health care services is well known as a predictor of morbidity. In a study like this, accessibility may play a confounding role especially while examining malpractice. At this point we should explain that in Israel we have a national health insurance law and all citizens are well insured. The accessibility to primary and consulting health care services is very high and does not confound our results.

In summary, despite some limitations, we offer here an efficient tool for analysis of decision making and quality assurance, which can be implemented in any health organization for management of any chronic disease.

## Authors' contributions

This article summarizes Ronit Peled's Ph.D thesis. Dr. Peled carried out the data collection, file management and data analysis. Prof. J.Pliskin, Prof. H.Reuveni and Prof. A. Tal were the Ph.D advisors and carried out the development of the "markers" system, assisted in the data analysis and supervised the whole process. Dr. Bennenson was the GIS advisor and with E.Hatna carried out the GIS mapping.
